# SILENT Syndrome – a Case of Lithium Neurotoxicity on Maintenance Therapy

**DOI:** 10.1192/bjo.2024.694

**Published:** 2024-08-01

**Authors:** Ailbhe Tummon, Elizabeth Walsh

**Affiliations:** University Hospital Galway, Galway, Ireland

## Abstract

**Aims:**

Lithium is licensed to treat bipolar disorder, which is characterized by recurrent episodes of depression and mania/hypomania. It is also used as an adjunctive medication in patients who have inadequately responded to first and second line treatments of unipolar depression. Lithium has a narrow therapeutic index and the potential for toxicity requires levels to be closely monitored, particularly during any intercurrent illness or initiation of new medications. There is a rare but important effect of lithium toxicity of which there is little awareness: the Syndrome of Irreversible Lithium Effectuated Neurotoxicity.

**Methods:**

A 57-year-old lady presented to the emergency department with a ten-day history of vomiting, diarrhoea, and abdominal pain. She had a history of recurrent depressive disorder managed with fluoxetine and lithium for ten years. On presentation, she was hypovolemic and required resuscitation with I.V. fluids. Clinical examination revealed significant ataxia and myoclonus. Neurological examination was limited by her inability to follow commands. She was orientated to person but not time or place. A collateral history was obtained from her husband. He reported a 3-day history of increasing confusion on a background of a 6-week history of gradual functional decline. History and examination were concerning for lithium neurotoxicity. Lithium level = 2.6mmol/L (0.4–0.8mmol/L), indicating lithium toxicity. Deterioration in renal function from baseline - urea 14.2, creatine level 120 umol/L eGFR 43mil/min. There was no evidence of infection, full blood count and CRP were within normal parameters. MRI brain showed mild degree global volume loss consistent with chronic small vessel microvascular ischaemia. She was commenced on haemodialysis in order to rapidly reduce her serum lithium levels.

**Results:**

Lithium levels post haemodialysis were 1.2mmol/L and within days fell to <0.4mmol/L. Further lithium treatment was held during admission, but she continued to exhibit signs of neurotoxicity. Two weeks post-admission her confusion persisted (MOCA 13/30). She remained tremulous and ataxic. A diagnosis of Syndrome of Irreversible lithium-effectuated neurotoxicity (SILENT) was made. She required intensive physiotherapy and occupational therapy input. 8 weeks post admission she had returned to her cognitive baseline and was mobilising independently.

**Conclusion:**

SILENT syndrome is a rare consequence of lithium toxicity secondary to elevated lithium levels in the central nervous system which if sustained it is thought can lead to cerebellar demyelination as was evidenced in this case by our patient's symptoms. The insidious onset of her lithium toxicity in the community led to a prolonged period of toxicity that went undetected. No clear precipitating factor was identified. Vigilance is required for toxicity and this case highlights the importance of family members being aware of the signs. A patient when confused may no longer be able to advocate clearly for themselves or seek appropriate medical attention. The patient also delayed consulting with her GP as her GP practice was located an hour and a half from her home and she could not secure an appointment during the summer months. Patients prescribed lithium require timely access to GPs for monitoring and consultations. Despite her experience with toxicity, the patient opted to restart lithium due to a recurrence of depressive symptoms. She is adhering to close monitoring of serum lithium levels. The patient and her family received thorough psycho-education regarding symptoms and signs of lithium toxicity.

